# Risk prediction models for dementia constructed by supervised principal component analysis using miRNA expression data

**DOI:** 10.1038/s42003-019-0324-7

**Published:** 2019-02-25

**Authors:** Daichi Shigemizu, Shintaro Akiyama, Yuya Asanomi, Keith A. Boroevich, Alok Sharma, Tatsuhiko Tsunoda, Kana Matsukuma, Makiko Ichikawa, Hiroko Sudo, Satoko Takizawa, Takashi Sakurai, Kouichi Ozaki, Takahiro Ochiya, Shumpei Niida

**Affiliations:** 1 Medical Genome Center, National Center for Geriatrics and Gerontology, Obu, Aichi, 474-8511 Japan; 2 Department of Medical Science Mathematics, Medical Research Institute, Tokyo Medical and Dental University (TMDU), Tokyo, 113-8510 Japan; 3 RIKEN Center for Integrative Medical Sciences, Yokohama, Kanagawa, 230-0045 Japan; 4 CREST, JST, Tokyo, 102-8666 Japan; 5 School of Engineering & Physics, University of the South Pacific, Suva, Fiji; 6 Institute for Integrated and Intelligent Systems, Griffith University, Brisbane, QLD, 4111 Australia; 7 Toray Industries, Inc., Kamakura, Kanagawa, 248-0036 Japan; 8 The Center for Comprehensive Care and Research on Memory Disorders, National Center for Geriatrics and Gerontology, Obu, Aichi, 474-8511 Japan; 9 Department of Cognitive and Behavioral Science, Nagoya University Graduate School of Medicine, Nagoya, Aichi, 466-8550 Japan; 10 Division of Molecular and Cellular Medicine, Fundamental Innovative Oncology Core Center, National Cancer Center Research Institute, Tokyo, 104-0045 Japan; 11 Institute of Medical Science, Tokyo Medical University, Tokyo, 160-8402 Japan

## Abstract

Alzheimer’s disease (AD) is the most common subtype of dementia, followed by Vascular Dementia (VaD), and Dementia with Lewy Bodies (DLB). Recently, microRNAs (miRNAs) have received a lot of attention as the novel biomarkers for dementia. Here, using serum miRNA expression of 1,601 Japanese individuals, we investigated potential miRNA biomarkers and constructed risk prediction models, based on a supervised principal component analysis (PCA) logistic regression method, according to the subtype of dementia. The final risk prediction model achieved a high accuracy of 0.873 on a validation cohort in AD, when using 78 miRNAs: Accuracy = 0.836 with 86 miRNAs in VaD; Accuracy = 0.825 with 110 miRNAs in DLB. To our knowledge, this is the first report applying miRNA-based risk prediction models to a dementia prospective cohort. Our study demonstrates our models to be effective in prospective disease risk prediction, and with further improvement may contribute to practical clinical use in dementia.

## Abstract (web summary)

Daichi Shigemizu et al. developed a risk prediction model using potential miRNA biomarkers of different dementias identified by a supervised principal component analysis logistic regression method. Their models achieved high accuracy when tested on a validation cohort and demonstrate the potential application of miRNA-based risk prediction models.

## Introduction

With an increasingly aging global human population, the number of people with dementia is rapidly increasing, and is estimated to reach 75 million by 2030 and 135 million by 2050, worldwide^1^. Since dementia is a clinical syndrome that leads to difficulties in daily activities involving memory, language and behavior, this rapid increase raises a substantial burden for medical care and public health systems^2^. On the other hand, there is no current cure for this disease, and the available treatments are only able to postpone the progression^3^. Therefore, identification of new biomarkers for earlier diagnosis and therapeutic intervention of the disease is promptly required^4^.

The diagnosis of dementia is generally based on the patients’ cognitive function^5^. Alzheimer’s disease (AD) is the most common subtype of dementia, followed by vascular dementia (VaD), and dementia with Lewy bodies (DLB)^1^. While recent studies have showed that three proteins in the cerebrospinal fluid (CSF): amyloid-beta 1–42 (Aβ_142_), total tau (T-tau) and phosphorylated tau 181 (P-tau_181_), could be effective in characterizing AD^6,7^, it is still challenging to use these CSF molecules as biomarkers in general physical examination for early diagnosis and therapeutic intervention due to the highly invasive collection process. In addition, new imaging-based techniques, including positron emission tomography scans for detection of amyloid-beta deposition or tau tracers, and the volumetric magnetic resonance imaging with determination of hippocampal or medial temporal lobe atrophy, are not suitable for initial screening due to the high cost performance^8–10^. It has also been reported that microRNAs play a key role in the control of glial cell development in the central nervous system^11^. Therefore, the present study is evaluated on the hypothesis that neurite and synapse destruction, associated with pathologic of dementia and other neurodegenerative diseases, can be detected in vitro by quantitative analysis of brain-enriched cell-free microRNA in the human blood^5^.

MicroRNAs (miRNAs) are approximately 22-nucleotide small non-coding RNAs, which have been shown to regulate gene expression by binding to complementary regions of messenger transcripts. The alteration of some miRNAs expression has recently been found in neurons of patients with AD and other neurodegenerative diseases^12–14^, and hence miRNAs are expected to be useful as easily accessible and non-invasive biomarkers^15^.

Here, we performed a comprehensive miRNA expression analysis using 1601 serum samples, composed of dementia patients and individuals with cognitive normal function (referred to as normal controls (NC)), in order to investigate new biomarkers for earlier diagnosis and therapeutic intervention and to construct risk prediction models using the biomarkers. We applied 10-fold cross-validation to a discovery cohort of 1092 individuals, separated from a validation cohort of 1089 individuals. We performed a two-step procedure similar to those used for risk prediction in several previous disease studies^16–19^. We first selected effective miRNA biomarker candidates in the logistic regression risk prediction models. Using the pre-selected miRNAs and the principal component scores (PC scores), we then constructed risk prediction models based on a supervised principal component analysis (PCA) logistic regression method. Finally, we determined the optimal miRNA and PC score set though cross-validation. This final risk prediction model, constructed based on the entire discovery cohort, was evaluated with an independent validation cohort by the area under the receiver operating characteristic curve (AUC). We further evaluated the predictive ability of our model using a prospective cohort. Our findings indicate that the prediction models using serum miRNA expression data may be useful as biomarkers for dementia and contribute to the development of future therapeutic measurement for this common but serious disorder.

## Results

### Japanese samples

We divided 1601 Japanese individuals (1021 AD cases, 91 VaD cases, 169 DLB cases, 32 mild cognitive impairment (MCI), and 288 NC) into a discovery cohort of 786 individuals (511 AD cases, 46 VaD cases, 85 DLB cases and 144 NC) and a validation cohort of 783 individuals (510 AD cases, 45 VaD cases, 84 DLB cases, and 144 NC) (see Materials and methods). The separation was performed to result in a similar distribution in the age between the discovery and validation cohorts for each disease (Table 1).

**Table 1:** Average age, sex and APOE information in the discovery and validation cohorts

	Discovery cohort				Validation cohort			
Phenotype	#Sample	Age	Sex (Male)	APOE^a^	#Sample	Age	Sex (Male)	APOE^a^
AD	511	79.2	0.29	0.53	510	79.2	0.31	0.47
VaD	46	79.0	0.63	0.33	45	79.1	0.56	0.18
DLB	85	79.5	0.45	0.34	84	79.5	0.36	0.30
NC	144	71.7	0.49	0.22	144	71.8	0.56	0.15

*APOE* apolipoprotein E, *AD* Alzheimer’s disease, *VaD* vascular dementia, *DLB* dementia with Lewy bodies, *NC* normal control

^a^APOE shows the average of the number of APOE ε_4_ allele genotype

### Construction of risk prediction models

**Fig. 1:**
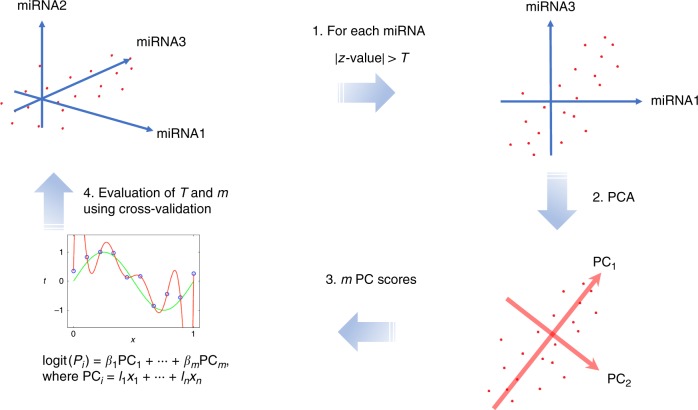
Workflow of our risk prediction model construction with supervised principal component analysis (PCA) logistic regression method. We calculated *z*-value in the logistic regression method for each microRNA (miRNA). The cutoff value of the *z*-value, *T* and the number of miRNAs, *n*, was pre-selected (1). The PCA was performed using the pre-selected miRNAs (2). The risk prediction models were constructed based on the combination of the miRNAs and *m* PC scores (3). This optimal parameter set (*T*, *m*) was determined in the discovery cohort using 10-fold cross-validation (4)

**Fig. 2:**
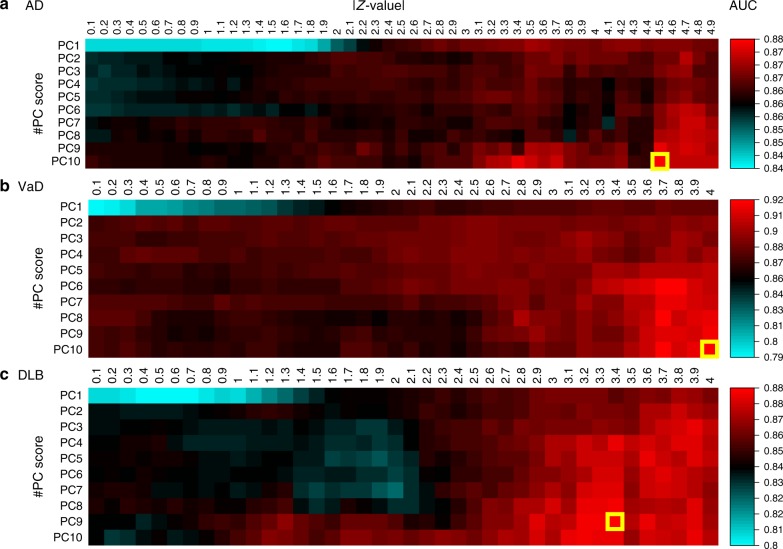
Risk prediction models using 10-fold cross-validation on a discovery cohort. The *x* and *y* axes show the cutoff value of the *z*-value in the logistic regression (*T*) and the number of top principal component (PC) scores used (*m*) for the prediction models, respectively. In AD (**a**), a combination of (*T*, *m*) = (4.5, 10) achieved the highest AUC of 0.877 in the discovery cohort. In VaD (**b**), a (*T*, *m*) = (4.0, 10) achieved an AUC = 0.923, and in DLB (**c**), a (*T*, *m*) = (3.4, 9) achieved an AUC = 0.885. AUC area under the curve, AD Alzheimer’s disease, VaD vascular dementia, DLB dementia with Lewy bodies

**Fig. 3:**
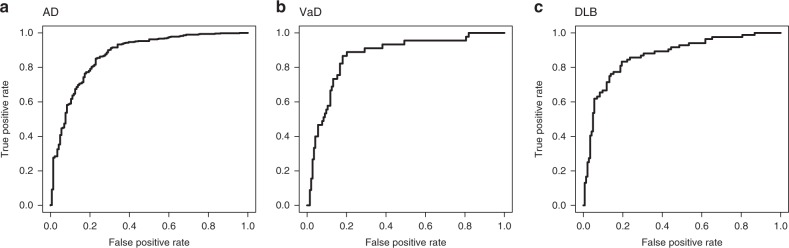
The ROC curves of our risk prediction models in a validation cohort. Final risk prediction models were constructed based on the supervised principal component analysis (PCA) logistic regression method in each disease using the complete discovery cohort. The adjusted models were then evaluated on the validation cohort. The final model construction in AD achieved an AUC of 0.874 in the validation cohort (**a**): AUC = 0.867 in VaD (**b**); AUC = 0.870 in DLB (**c**). Sensitivity and specificity were maximized at a sensitivity of 0.933 and specificity of 0.660 in AD (**a**): a sensitivity of 0.733 and specificity of 0.868 in VaD (**b**); and a sensitivity of 0.762 and specificity of 0.861 in DLB (**c**). ROC receiver operating characteristic, AUC area under the curve, AD Alzheimer’s disease, VaD vascular dementia, DLB dementia with Lewy bodies

Our risk prediction models were constructed based on a supervised PCA logistic regression method. All approaches that we considered were carried out on datasets of the *p* pre-selected miRNAs (*p* ≤ 2562). The selection of miRNAs was carried out based on the *z*-value in the logistic regression. Nine-tenths of entire training set was used for the calculation of the *z*-values and to fit the model for each cross-validation step. The adjusted model was evaluated using the remaining one-tenth of the training set. This process was repeated 10 times (10-fold cross-validation). The cutoff value *T* of the *z*-values was then raised from 0.1 to 5.0 at an interval 0.1. The number of top PC scores used, *m*, was set from 1 to 10 (Fig. 1). On the basis of the average AUC, we investigated all combinations of the *T* and *m*, and in AD, the combination of (*T*, *m*) = (4.5, 10) achieved the highest AUC of 0.877 in the discovery cohort. In VaD, a (*T*, *m*) = (4.0, 10) achieved an AUC = 0.923, and in DLB, a (*T*, *m*) = (3.4, 9) achieved an AUC = 0.885 (Fig. 2). Final risk prediction models were constructed based on the optimal *T* and *m* detected in each disease using the entire training set (discovery cohort). The adjusted models were then evaluated on the validation cohort, which was completely independent from the discovery cohort. As a result, 78 miRNAs out of 2562 were employed for the final model construction in AD, which achieved an AUC of 0.874 in the validation cohort (Fig. 3a). Of the 78, two miRNAs (MIMAT0004947 and MIMAT0022726) were AD-specific miRNAs reported in previous studies^20^. The remaining previously reported miRNAs did not show significantly better outcome in logistic regression in the selection of miRNAs (Supplementary Data 1). A maximum average sensitivity and specificity of the ROC curve was achieved at a sensitivity of 0.933 and specificity of 0.660 in AD. The accuracy showed 0.873 when the prognostic index was 0.281 (Table 2). In a similar way, 86 miRNAs and 110 miRNAs were employed for our final model construction in VaD (Fig. 3b) and DLB (Fig. 3c), which achieved AUCs of 0.867 and 0.870 in the validation cohort, respectively. A maximum average sensitivity and specificity of the ROC curve was achieved at a sensitivity of 0.733 and specificity of 0.868 in VaD and at a sensitivity of 0.762 and specificity of 0.861 in DLB. The accuracies in VaD and DLB were 0.836 and 0.825 when the prognostic index was −0.761 and 0.0392, respectively (Table 2).

**Table 2:** Accuracy estimation in three diseases using the validation cohort

Disease	PI cutoff	Accuracy	Sensitivity	Specificity
AD	0.281	0.873	0.933	0.660
VaD	−0.761	0.836	0.733	0.868
DLB	0.0392	0.825	0.762	0.861

*PI* prognostic index, *AD* Alzheimer’s disease, *VaD* vascular dementia, *DLB* dementia with Lewy bodies

We also constructed risk prediction models based on the logistic regression method using only clinical information (age, sex and apolipoprotein E (APOE)) for the entire training data set. The adjusted models were then evaluated on the validation cohort. The AUCs achieved were 0.857 in AD, 0.813 in VaD and 0.827 in DLB. Our finding’s miRNAs contributed to an increase of AUCs for the risk prediction models. We further compared our two-step method with a one-step penalized regression method, LASSO (least absolute shrinkage and selection operator). We constructed risk prediction models based on the LASSO method using all miRNAs in the entire training data set. The adjusted models were then evaluated on the validation cohort. The AUCs achieved were 0.898 in AD, 0.821 in VaD and 0.892 in DLB. For AD and DLB, the one-step penalized regression method showed similar AUCs to our two-step method, but the penalized method showed a lower AUC in VaD than our method (LASSO = 0.821, our method = 0.867).

### Effective miRNAs and the functional gene annotations

**Fig. 4:**
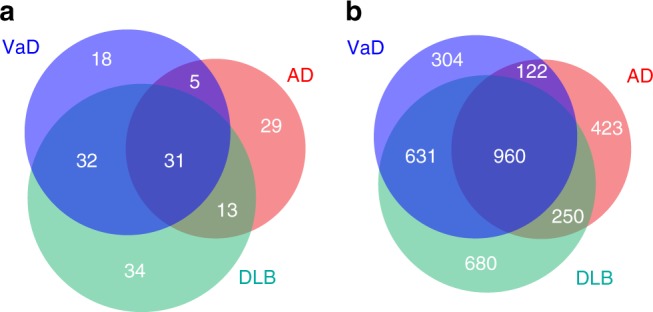
Effective microRNAs (miRNAs) and genes used in risk prediction model. **a** The number of miRNAs used for final risk prediction models were 78, 86 and 110 in AD, VaD and DLB, respectively. The pie-chart showed the common and disease-specific miRNAs among these three diseases. **b** Using microRNA Target Prediction and Functional Study Database (miRDB), which can predict miRNA functional target genes, the 78 miRNAs in AD were predicted to target 1755 genes. The 86 miRNAs in VaD and 110 miRNAs in DLB were predicted to target 2017 and 2521 genes, respectively. The pie-chart showed the common and disease-specific target genes among these three diseases. AD Alzheimer’s disease, VaD vascular dementia, DLB dementia with Lewy bodies

The number of miRNAs used for final risk prediction models were 78, 86 and 110 in AD, VaD and DLB, respectively (Supplementary Data 2). We next examined the common and disease-specific miRNAs among these three diseases. A large number of miRNAs were shared between VaD and DLB (32 miRNAs) and among all three (31 miRNAs) (Fig. 4a). AD possessed the most disease-specific miRNAs compared to the other diseases (AD; 29/78 = 0.371, DLB; 34/110 = 0.309, VaD; 18/86 = 0.209) (Fig. 4a).

Overall, miRNAs regulate the expression of thousands of protein-coding gene targets (mRNAs) at both post-transcriptional and translational levels^21–23^. To determine the biological significance of our findings (miRNAs), we examined microRNA Target Prediction and Functional Study Database (miRDB^24^), which can predict miRNA functional target genes. The 78 miRNAs in AD were predicted to target 1755 genes. In the similar way, the 86 miRNAs in VaD and 110 miRNAs in DLB were predicted to target 2017 and 2521 genes, respectively. Compared with miRNAs, a large number of mRNAs were shared among three diseases (miRNAs: 31/162 = 0.191, mRNAs: 960/3370 = 0.285) (Fig. 4a, b).

### Functional modules using co-expression network analysis

**Fig. 5:**
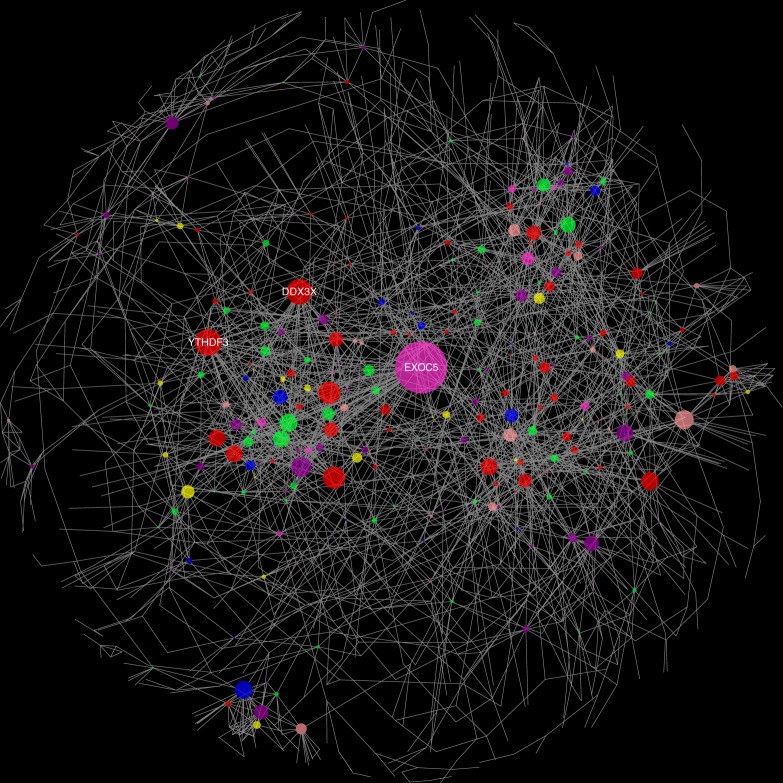
Gene co-expression network analysis. Node size corresponds to the number of connected edges. The gene name is displayed for nodes with >25 edges. Node color corresponds to which diseases the gene is associated: AD (orange), VaD (blue), DLB (green), AD and VaD (pink), AD and DLB (yellow), VaD and DLB (purple), and all three (red). AD Alzheimer’s disease, VaD vascular dementia, DLB dementia with Lewy bodies

Since we detected several candidate gene targets in the three diseases, we next attempted to elucidate functional modules from the candidates. We focused on the occurrence of hub genes, which have relationships with many genes, through large-scale gene co-expression network analysis. The gene co-expression information was gathered from the COXPRESdb database^25^ (see Materials and methods). Gene co-expression network visualization was performed using Cytoscape software^26^. Three hub genes, which co-expressed with >25 genes, were detected in the functional modules (*EXOC5*, *DDX3X* and *YTHDF3*, Fig. 5). *EXOC5* was associated with AD and VaD, and the remaining two genes were common among the three diseases. These three genes were also verified to express in brain tissue through the Genotype-Tissue Expression (GTEx) project^27,28^.

### Validation in a prospective cohort

We measured miRNA expression for 32 MCI subjects, which were obtained from the prospective data, of which 10 subjects converted to AD after at least 6 months. We evaluated if our risk prediction model in AD could predict the converted subjects after 6 months. Prognosis indices (PIs) assigned to each subject were calculated by applying 78 miRNA expression values to our prediction model. A PI score greater than 0.281 predicted the subject would convert to AD (Table 2). As a result, all of the 10 converted subjects were correctly predicted by our model (sensitivity = 1.0). Furthermore, all 4 subjects predicted not to covert to AD did not actually convert to AD (negative predictive value = 1.0). The remaining 18 subjects were predicted to convert to AD, but had not yet converted (specificity = 0.18) (Table 3). For further validation of this discordance, we may have to follow-up with the subjects in the future and use the additional comprehensive information, including genetic data and/or whole transcriptome data, for further improvement for practical clinical use in dementia.

**Table 3:** Validation using the prospective cohort

		Prospective cohort		
	Conversion	MCI to AD	MCI to MCI	Total
Prediction	MCI to AD	10	18	28
	MCI to MCI	0	4	4
	Total	10	22	32

*MCI* mild cognitive impairment, *AD* Alzheimer’s disease

## Discussion

New biomarkers for early diagnosis and intervention have been examined in many diseases^29–31^. The role of serum miRNAs has recently been reviewed with emphasis on their impact on the etiopathogenesis of sporadic AD^32^ and cancers^21–23,33,34^. For example, dysregulated serum miRNAs, such as the downregulation of miR-137, miR-181c, miR-9, and miR-29a/b in the blood of AD patients, have been identified^35–38^. It has also been reported that expressional differences between AD and other dementia types were observed for some miRNAs^39^. However, due to the small sample sizes in these previous reports, comprehensive miRNA expression analysis had not been performed in AD or the other subtypes of dementia. Therefore, in this study we investigated biomarkers with respect to each subtype of dementia, using serum miRNAs and a larger sample size.

We first detected optimal parameters for risk prediction models using cross-validation of a discovery cohort. The final models were then constructed with the optimal parameters using the complete discovery cohort. The adjusted models were finally evaluated on an independent validation cohort using AUC as the discriminative accuracy of these risk prediction models. In general, these risk prediction models on cross-validation of the discovery cohort achieved higher AUCs than the adjust models on the validation cohort^18,40^. The difference of the AUCs is due to overfitting of the model construction criterion. However, our risk models showed only small differences between discovery and validation cohorts in the AUCs (AD = 0.877 and 0.874, VaD = 0.923 and 0.867, and DLB = 0.885 and 0.870). These results imply the miRNAs used in our models were efficient to classify disease samples and non-disease samples, although additional replication studies are necessary in future work. We also constructed the risk prediction models using a larger maximum value of PC scores (*m* = 50). In AD and DLB, the AUCs of the final models were slightly increased in a validation cohort in the combination of (*T*, *m*) = (3.6, 41) in AD and that of (*T*, *m*) = (3.2, 12) in DLB, compared with those in *m* = 10 (AD = + 0.007, DLB = +0.002, Supplementary Table 1), but that in VaD was considerably decreased in the combination of (*T*, *m*) = (3.8, 13) (VaD = −0.015, Supplementary Table 1). For all, a larger number of miRNAs were required for the final model construction: *m* = 50 (AD, VaD, DLB) = (171, 134, 143) miRNAs, *m* = 10 (AD, VaD, DLB) = (78, 86, 110) miRNAs (Supplementary Table 1). When considering prediction models with a low number of biomarkers, our approaches would be efficient for optimal risk prediction models. We further compared our final models using pre-selected miRNAs to those using all miRNAs. Our models using pre-selected miRNAs had superior AUCs to those using all miRNAs in all three diseases for both *m* = 10 and *m* = 50 (Supplementary Table 2). Investigations using larger sample sizes will lead to further improvement in the performance of risk prediction models.

The annotation of gene targets for miRNAs is critical for functional characterization of our findings. We used miRDB^24^ for these functional gene annotation from miRNAs. A large number of genes associated with the dementia was detected. We further elucidated three functionally important modules (i.e. hub genes, *EXOC5*, *DDX3X* and *YTHDF3*) through large-scale gene co-expression network analysis. These three genes were verified to express in brain tissue through the GTEx project^27,28^. Jun et al.^41^ have reported that a single-nucleotide polymorphism (SNP) in the *EXOC5* showed evidence for association with AD. *DDX3X*, the DEAD (Asp-Glu-Ala-Asp) box helicase 3, X-linked, belongs to ATP-dependent RNA helicase, the activation of which is associated with cancer in many tissues, including brain^42–44^. Previous studies have reported that *DDX3X* expression level is positively correlated with poor survival outcome in human glioma^45^. Also, several studies have reported that YTHDF protein could be associated with accumulation of m6A-modified transcripts^46^, and this m6A mRNA modification is critical for glioblastoma stem cell self-renewal and tumorigenesis^47^. Furthermore, recent transcriptomic meta-analyses revealed that AD and glioblastoma patients had similar expression patterns in a number of genes^48^. These observations support the existence of molecular substrates that could partially account for direct co-morbidity relationships^49–51^. These results suggest that the three hub genes detected could not only play a key role in pathogenesis of dementia, but also contribute to discovery of novel drug targets.

The diagnosis of dementia is not always consistent with brain pathological changes^52,53^. Also, elderly dementia patients often have concomitant cerebrovascular disease pathologies as well as other concomitant neurodegenerative disease pathologies^54^. We proposed a methodology that finds the best risk prediction model for each disease rather than a general model that could be applied to any data set. Our proposed models might be able to differentiate these complex neurological disorders. However, further refinement of this methodology will be required before its practical use in healthcare. One way may be to consider genetic variations, such as SNPs and insertions and deletions (indels) and gene expressions. The development of next-generation sequencing technology has facilitated comprehensive analysis of these genetic and expression data. There is no doubt that these additional data would contribute to further improvement of our risk prediction models.

## Materials and methods

### Ethics statements

This study was approved by the ethics committee of the National Center for Geriatrics and Gerontology (NCGG). The design and performance of current study involving human subjects were clearly described in a research protocol. All participants were voluntary and completed informed consent in writing before registering to NCGG Biobank.

### Clinical samples

All 1601 serum subjects and the associated clinical data were distributed from the NCGG Biobank, which collects human biomaterials and data for geriatrics research. Of them, 1021 subjects were patients with AD: 91 patients with VaD, 169 patients with DLB, 32 patients with MCI and 288 subjects were normal controls with normal cognitive function (NC). NCs who had subjective cognitive complaints, but normal cognition on the neuropsychological assessment, were categorized as normal controls. The AD and MCI subjects were diagnosed with a probable or possible AD based on the criteria of the National Institute on Aging Alzheimer’s Association workgroups^55,56^. We used the probable ADs as AD subjects in this study. The VaD and DLB subjects were diagnosed based on the criteria of report of the NINDS-AIREN International Workshop^57^ and fourth report of the DLB Consortium^58^, respectively. The diagnosis of all subjects was conducted based on medical history, physical examination and diagnostic tests, neurological examination, neuropsychological tests and brain imaging with magnetic resonance imaging or computerized tomography by experts including neurologists, psychiatrists, geriatricians or a neurosurgeon, all experts in dementia who are familiar with its diagnostic criteria. Comprehensive neuropsychological tests included Mini-Mental State Examination (MMSE), Alzheimer’s Disease Assessment Scale Cognitive Component Japanese version, Logical Memory I and II from the Wechsler Memory Scale–Revised, frontal assessment battery, Raven’s colored progressive matrices and Geriatric Depression Scale^59^. If necessary, dopamine transporter imaging and metaiodobenzylguanidine myocardial scintigraphy were performed for the diagnosis of DLB. Pathological tests and biomarkers in cerebrospinal fluid tests were not used for the diagnosis of dementia. For all of the subjects, the status of the APOE ε_4_ allele genotype (the major genetic risk factor with AD) and the MMSE score were obtained. All subjects were >60 years in age. All NC subjects had a MMSE score of >23.

### miRNA expression

Serum samples were isolated from whole blood following the standard operating procedure of NCGG Biobank. In brief, blood samples tubes were gently inverted a few times, put in an upright-position for at least 30 min to clot, and then centrifuged for 15 min at 3500 rpm at 4 °C. After centrifugation, serum was transferred to storage tubes containing 500 μl per tube and immediately stored in −80 °C freezers. Total RNA was extracted from a 300 μl serum sample using a 3D‐Gene RNA extraction reagent from a liquid sample kit (Toray Industries, Inc.), as previously described^34^. Comprehensive miRNA expression analysis was performed using a 3D‐Gene miRNA Labeling kit and a 3D‐Gene Human miRNA Oligo Chip (Toray Industries, Inc.), which was designed to detect 2562 miRNA sequences registered in miRBase release 21 (http://www.mirbase.org/).

The normalization of miRNA expression was performed by the following steps. Mean and standard deviation (SD) were calculated using a set of pre-selected negative control signals (background signals), the top and bottom 5% of which were removed. Signal values greater than mean + 2 SD of the background signals were replaced using log2(signal–mean) and labeled effective signals. The remaining signal values were replaced by the minimum of the effective signals–0.1. Undetected signal values were replaced by the average signal of each miRNA signal. To normalize the signals across different microarrays, a set of pre-selected internal control miRNAs (miR-149-3p, miR-2861 and miR-4463), which had been stably detected in more than 500 serum samples, was used. Each miRNA signal value was standardized with the ratio of the average signal of the three internal control miRNA signals^34^.

### Risk prediction model construction

We calculated the *z*-value corresponding to the miRNA in the logistic regression model in each disease (AD, VaD and DLB, Fig. 1) in the following way:

$${\mathrm{logit}}\left({P}_{i}\right) = \alpha _{0} + \alpha _{1} \times {\mathrm {Sex}}_{i} + \alpha _{2} \times {\mathrm {Age}}_{i} + \alpha _{3} \times {\mathrm {APOE}}_{i} + \alpha _{4} \times {\mathrm {miRNA}}_{i},$$

The *z*-value was the regression coefficient divided by its standard error. The cutoff value, *T*, of the *z*-value, and *n*, the number of miRNAs (*n* = 1, …, 2562), was pre-selected (Fig. 1). Next, the PCA was performed using the pre-selected miRNAs. The risk prediction models were constructed based on the combination of the miRNAs and PC scores as defined by Fig. 1:

$${\mathrm {logit}}\left( {{P}_{i}} \right) = \beta _1 \times {\mathrm {PC}}_1 + \ldots + \beta _{m} \times {\mathrm {PC}}_{m},$$

where PC_*i*_ = *l*_1_ × *x*_1_ + … + *l*_*n*_ × *x*_*n*_, and *x*_*j*_ is the normalized expression value of miRNA_*j*_. These calculations were iteratively performed for all combinations of cutoff values (*T* = 0.1, 0.2, …, 5.0) and the top PC scores (*m* = 1, …,10) (Fig. 1). This optimal parameter set (*T, m*) was determined in the discovery cohort using 10-fold cross-validation. The regression method used in this study was conducted using the *glmnet* package in the statistical software R^60^.

### Evaluation of risk prediction models

All data were strictly separated into the discovery cohort and validation cohort. An optimal parameter set (*T, m*) was detected using 10-fold cross-validation in the discovery cohort with respect to each disease (AD, VaD and DLB). Final models were constructed with the optimal parameter sets using the complete discovery cohort. The adjusted models were evaluated on an independent validation cohort. The receiver operator characteristic (ROC) curves^61^ on the validation cohort and the AUC were used as the discriminative accuracy of the risk prediction models. In order to further apply these final risk prediction models to prospective cohort data, we calculated prognostic index in each sample as defined by:

$${\mathrm {prognostic}\, {\mathrm {index}}} = \mathop {\sum }\limits_i \beta _i \times PC_i \,,$$

where *β* is the estimated regression coefficient of each PC score using a supervised PCA logistic regression method in the discovery cohort. These optimal prognostic indices were determined using a maximum average sensitivity and specificity of the ROC curve in the discovery cohort.

### Target gene annotation of miRNAs

The functional gene annotation of miRNAs was conducted using miRDB, which includes predicted gene targets regulated by a comprehensive 6709 miRNAs^24^. All the gene targets have a prediction score in the range between 0 and 100 assigned by MirTarget V3, with a higher score representing more statistical confidence in the prediction result. Only gene targets with the score of >90 were used as functional gene annotation for our analysis.

### Gene co-expression network analysis

COXPRESdb^25^ provides gene co-expression relationships for 11 animal species (human, mouse, rat, monkey, dog, chicken, zebrafish, fly, nematode, budding yeast and fission yeast). For all gene pairs, Pearson’s correlation coefficients were calculated, and these values were transferred to the Mutual Rank (MR) value^62^, which is the geometric average of asymmetric ranks in co-expressed gene lists. In this study, gene pairs with a MR < 20 and Pearson’s correlation coefficients > 0.4 in human were used as co-expression genes. The gene co-expression network was generated using Cytoscape v3.5.1^26^.

### Code availability

We used open source program languages R (version 3.4.1), Ruby (version 2.4.0) and Python (version 3.5.1) to analyze data and create plots. Code is available upon request from the corresponding authors.

### Reporting summary

Further information on experimental design is available in the Nature Research Reporting Summary linked to this article.

## Supplementary information

Supplementary Information

Reporting Summary

Supplementary Data 1

Supplementary Data 2

Description of Additional Supplementary Files

## Data Availability

All microarray data (2562 miRNAs) of this study are publicly available through the Gene Expression Omnibus (GEO) database at the National Center for Biotechnology Information (NCBI) and accessible through GEO series accession number GSE120584. Datasets generated during the current study are available from the corresponding author on reasonable request.

## References

[1] Robinson L, Tang E, Taylor JP (2015). Dementia: timely diagnosis and early intervention. BMJ.

[2] Haan MN, Wallace R (2004). Can dementia be prevented? Brain aging in a population-based context. Annu. Rev. Public Health.

[3] Kim DH (2014). Genetic markers for diagnosis and pathogenesis of Alzheimer’s disease. Gene.

[4] Spires-Jones TL, Hyman BT (2014). The intersection of amyloid beta and tau at synapses in Alzheimer’s disease. Neuron.

[5] Sheinerman KS (2012). Plasma microRNA biomarkers for detection of mild cognitive impairment. Aging.

[6] Fagan AM (2011). Comparison of analytical platforms for cerebrospinal fluid measures of beta-amyloid 1-42, total tau, and p-tau181 for identifying Alzheimer disease amyloid plaque pathology. Arch. Neurol..

[7] De Meyer G (2010). Diagnosis-independent Alzheimer disease biomarker signature in cognitively normal elderly people. Arch. Neurol..

[8] Mistur R (2009). Current challenges for the early detection of Alzheimer’s disease: brain imaging and CSF studies. J. Clin. Neurol..

[9] Miller G (2009). Alzheimer’s biomarker initiative hits its stride. Science.

[10] Schmand B, Eikelenboom P, van Gool WA (2011). Value of neuropsychological tests, neuroimaging, and biomarkers for diagnosing Alzheimer’s disease in younger and older age cohorts. J. Am. Geriatr. Soc..

[11] Zheng K, Li H, Huang H, Qiu M (2012). MicroRNAs and glial cell development. Neuroscientist.

[12] Satoh J (2010). MicroRNAs and their therapeutic potential for human diseases: aberrant microRNA expression in Alzheimer’s disease brains. J. Pharmacol. Sci..

[13] Cogswell JP (2008). Identification of miRNA changes in Alzheimer’s disease brain and CSF yields putative biomarkers and insights into disease pathways. J. Alzheimers Dis..

[14] Tacutu R, Budovsky A, Yanai H, Fraifeld VE (2011). Molecular links between cellular senescence, longevity and age-related diseases - a systems biology perspective. Aging.

[15] Femminella GD, Ferrara N, Rengo G (2015). The emerging role of microRNAs in Alzheimer’s disease. Front. Physiol..

[16] Bair E, Tibshirani R (2004). Semi-supervised methods to predict patient survival from gene expression data. PLoS Biol..

[17] Kooperberg C, LeBlanc M, Obenchain V (2010). Risk prediction using genome-wide association studies. Genet. Epidemiol..

[18] Shigemizu D (2014). The construction of risk prediction models using GWAS data and its application to a type 2 diabetes prospective cohort. PLoS One.

[19] Liang Y (2016). Cancer survival analysis using semi-supervised learning method based on Cox and AFT models with L1/2 regularization. BMC Med. Genom..

[20] Wu HZ (2016). Circulating microRNAs as biomarkers of Alzheimer’s disease: a systematic review. J. Alzheimers Dis..

[21] Heneghan HM, Miller N, Kelly R, Newell J, Kerin MJ (2010). Systemic miRNA-195 differentiates breast cancer from other malignancies and is a potential biomarker for detecting noninvasive and early stage disease. Oncologist.

[22] Asaga S (2011). Direct serum assay for microRNA-21 concentrations in early and advanced breast cancer. Clin. Chem..

[23] Roth C (2010). Circulating microRNAs as blood-based markers for patients with primary and metastatic breast cancer. Breast Cancer Res..

[24] Wong N, Wang X (2015). miRDB: an online resource for microRNA target prediction and functional annotations. Nucleic Acids Res..

[25] Okamura Y (2015). COXPRESdb in 2015: coexpression database for animal species by DNA-microarray and RNAseq-based expression data with multiple quality assessment systems. Nucleic Acids Res..

[26] Shannon P (2003). Cytoscape: a software environment for integrated models of biomolecular interaction networks. Genome Res..

[27] Consortium GT (2013). The Genotype-Tissue Expression (GTEx) project. Nat. Genet..

[28] Consortium GT (2015). Human genomics. The Genotype-Tissue Expression (GTEx) pilot analysis: multitissue gene regulation in humans. Science.

[29] Fang C (2012). Serum microRNAs are promising novel biomarkers for diffuse large B cell lymphoma. Ann. Hematol..

[30] Mitchell PS (2008). Circulating microRNAs as stable blood-based markers for cancer detection. Proc. Natl. Acad. Sci. USA.

[31] Mizuno H (2011). Identification of muscle-specific microRNAs in serum of muscular dystrophy animal models: promising novel blood-based markers for muscular dystrophy. PLoS One.

[32] Maes OC, Chertkow HM, Wang E, Schipper HM (2009). MicroRNA: implications for Alzheimer disease and other human CNS disorders. Curr. Genom..

[33] Zhu W, Qin W, Atasoy U, Sauter ER (2009). Circulating microRNAs in breast cancer and healthy subjects. BMC Res. Notes.

[34] Shimomura A (2016). Novel combination of serum microRNA for detecting breast cancer in the early stage. Cancer Sci..

[35] Geekiyanage H, Jicha GA, Nelson PT, Chan C (2012). Blood serum miRNA: non-invasive biomarkers for Alzheimer’s disease. Exp. Neurol..

[36] Bekris LM (2013). MicroRNA in Alzheimer’s disease: an exploratory study in brain, cerebrospinal fluid and plasma. Biomarkers.

[37] Kumar P (2013). Circulating miRNA biomarkers for Alzheimer’s disease. PLoS One.

[38] Kiko T (2014). MicroRNAs in plasma and cerebrospinal fluid as potential markers for Alzheimer’s disease. J. Alzheimers Dis..

[39] Sorensen SS, Nygaard AB, Christensen T (2016). miRNA expression profiles in cerebrospinal fluid and blood of patients with Alzheimer’s disease and other types of dementia - an exploratory study. Transl. Neurodegener..

[40] Shigemizu D (2017). The prediction models for postoperative overall survival and disease-free survival in patients with breast cancer. Cancer Med..

[41] Jun G (2011). Genome-wide scan suggested novel Alzheimer’s disease susceptibility genes by factoring influence of APOE. J. Alzheimer’s Assoc..

[42] Bol GM (2013). Expression of the RNA helicase DDX3 and the hypoxia response in breast cancer. PLoS One.

[43] Wu DW (2014). DDX3 loss by p53 inactivation promotes tumor malignancy via the MDM2/Slug/E-cadherin pathway and poor patient outcome in non-small-cell lung cancer. Oncogene.

[44] Sun M, Song L, Zhou T, Gillespie GY, Jope RS (2011). The role of DDX3 in regulating Snail. Biochim. Biophys. Acta.

[45] Hueng DY (2015). DDX3X biomarker correlates with poor survival in human gliomas. Int. J. Mol. Sci..

[46] Shi H (2017). YTHDF3 facilitates translation and decay of N(6)-methyladenosine-modified RNA. Cell Res..

[47] Cui Q (2017). m(6)A RNA methylation regulates the self-renewal and tumorigenesis of glioblastoma stem cells. Cell Rep..

[48] Sanchez-Valle J (2017). A molecular hypothesis to explain direct and inverse co-morbidities between Alzheimer’s disease, glioblastoma and lung cancer. Sci. Rep..

[49] Lehrer S (2010). Glioblastoma and dementia may share a common cause. Med. Hypotheses.

[50] Driver JA (2012). Inverse association between cancer and Alzheimer’s disease: results from the Framingham Heart Study. BMJ.

[51] Musicco M (2013). Inverse occurrence of cancer and Alzheimer disease: a population-based incidence study. Neurology.

[52] Kosunen O (1996). Diagnostic accuracy of Alzheimer’s disease: a neuropathological study. Acta Neuropathol..

[53] Dubois B (2007). Research criteria for the diagnosis of Alzheimer’s disease: revising the NINCDS-ADRDA criteria. Lancet Neurol..

[54] Kapasi A, DeCarli C, Schneider JA (2017). Impact of multiple pathologies on the threshold for clinically overt dementia. Acta Neuropathol..

[55] McKhann GM (2011). The diagnosis of dementia due to Alzheimer’s disease: recommendations from the National Institute on Aging-Alzheimer’s Association workgroups on diagnostic guidelines for Alzheimer’s disease. Alzheimers Dement..

[56] Albert MS (2011). The diagnosis of mild cognitive impairment due to Alzheimer’s disease: recommendations from the National Institute on Aging-Alzheimer’s Association workgroups on diagnostic guidelines for Alzheimer’s disease. Alzheimers Dement..

[57] Roman GC (1993). Vascular dementia: diagnostic criteria for research studies. Report of the NINDS-AIREN International Workshop. Neurology.

[58] McKeith IG (2017). Diagnosis and management of dementia with Lewy bodies: fourth consensus report of the DLB Consortium. Neurology.

[59] Kawai Y (2013). Neuropsychological differentiation between Alzheimer’s disease and dementia with Lewy bodies in a memory clinic. Psychogeriatrics.

[60] R Development Core Team. *R: A Language and Environment for Statistical Computing* (R Foundation for Statistical Computing, Vienna, 2009).

[61] Sing T, Sander O, Beerenwinkel N, Lengauer T (2005). ROCR: visualizing classifier performance in R. Bioinformatics.

[62] Obayashi T, Kinoshita K (2009). Rank of correlation coefficient as a comparable measure for biological significance of gene coexpression. DNA Res..

