# Supplementary material for: Risk prediction models for dementia constructed by supervised principal component analysis using miRNA expression data
Source: Commun Biol. 2019 Feb 25;2:77. doi: 10.1038/s42003-019-0324-7 (PMC6389908; doi:10.1038/s42003-019-0324-7)
Supplement: Supplementary file 1 — Supplementary Information [file 42003_2019_324_MOESM1_ESM.pdf]

Supplementary Table 1. Risk prediction models using larger maximum value of PC scores (m=50)

Max #PCs	disease	*T	†m	#miRNAs	AUC	
					discovery cohort	validation cohort
10	AD	4.5	10	78	0.877	0.874
	VaD	4	10	86	0.923	0.867
	DLB	3.4	9	110	0.885	0.870
50	AD	3.6	41	171	0.900	0.881
	VaD	3.8	13	134	0.935	0.852
	DLB	3.2	12	143	0.893	0.872

*T: cutoff value of the z-value in the logistic regression

†m: the number of top PC scores used for the prediction models

Supplementary Table 2. Risk prediction models using all miRNAs

		Using pre-selected miRNAs		Using all miRNAs	
Max #PCs	disease	†m	AUC	†m	AUC
10	AD	10	0.877	10	0.862
	VaD	10	0.923	8	0.876
	DLB	9	0.885	10	0.846
50	AD	41	0.900	48	0.883
	VaD	14	0.920	8	0.876
	DLB	12	0.893	20	0.863

†m: the number of top PC scores used for the prediction models
