# Supplementary material for: Risk prediction models for dementia constructed by supervised principal component analysis using miRNA expression data
Source: Commun Biol. 2019 Feb 25;2:77. doi: 10.1038/s42003-019-0324-7 (PMC6389908; doi:10.1038/s42003-019-0324-7)
Supplement: Supplementary file 2 — Reporting Summary [file 42003_2019_324_MOESM2_ESM.pdf]

nature research

Corresponding author(s): Daichi Shigemizu

Last updated by author(s): Jan 12, 2019

Reporting Summary

Nature Research wishes to improve the reproducibility of the work that we publish. This form provides structure for consistency and transparency in reporting. For further information on Nature Research policies, see Authors & Referees and the Editorial Policy Checklist.

Statistics

For all statistical analyses, confirm that the following items are present in the figure legend, table legend, main text, or Methods section.

n/a	Confirmed	
☐	☒	The exact sample size (*n*) for each experimental group/condition, given as a discrete number and unit of measurement
☐	☒	A statement on whether measurements were taken from distinct samples or whether the same sample was measured repeatedly
☐	☒	The statistical test(s) used AND whether they are one- or two-sided<br>*Only common tests should be described solely by name; describe more complex techniques in the Methods section.*
☐	☒	A description of all covariates tested
☐	☒	A description of any assumptions or corrections, such as tests of normality and adjustment for multiple comparisons
☐	☒	A full description of the statistical parameters including central tendency (e.g. means) or other basic estimates (e.g. regression coefficient) AND variation (e.g. standard deviation) or associated estimates of uncertainty (e.g. confidence intervals)
☐	☒	For null hypothesis testing, the test statistic (e.g. *F*, *t*, *r*) with confidence intervals, effect sizes, degrees of freedom and *P* value noted<br>*Give P values as exact values whenever suitable.*
☒	☐	For Bayesian analysis, information on the choice of priors and Markov chain Monte Carlo settings
☐	☒	For hierarchical and complex designs, identification of the appropriate level for tests and full reporting of outcomes
☒	☐	Estimates of effect sizes (e.g. Cohen's *d*, Pearson's *r*), indicating how they were calculated

Our web collection on statistics for biologists contains articles on many of the points above.

Software and code

Policy information about availability of computer code

Data collection	The functional gene annotation data of miRNAs were collected from miRDB. The co-expression gene data in human were collected from COXPRESdb.
Data analysis	The miRNA expression was normalized using the open source program language R (version 3.4.1). Risk prediction model construction with supervised PCA logistic regression method was performed using the glmnet 2.0-10 R package. Evaluation of risk prediction models was performed using the pROC R package. The gene co-expression network was generated using Cytoscape v3.5.1. Figures were drawn in Adobe Illustrator CC and Microsoft 2016 for Mac.

For manuscripts utilizing custom algorithms or software that are central to the research but not yet described in published literature, software must be made available to editors/reviewers. We strongly encourage code deposition in a community repository (e.g. GitHub). See the Nature Research guidelines for submitting code & software for further information.

Data

Policy information about availability of data

All manuscripts must include a data availability statement. This statement should provide the following information, where applicable:

- Accession codes, unique identifiers, or web links for publicly available datasets
- A list of figures that have associated raw data
- A description of any restrictions on data availability

All microarray data (2,562 miRNAs) of this study are publicly available through the Gene Expression Omnibus (GEO) database at the National Center for Biotechnology Information (NCBI) and accessible through GEO series accession number GSE120584 at http://www.ncbi.nlm.nih.gov/projects/geo/. Datasets generated during the current study are available from the corresponding author on reasonable request.

Field-specific reporting

Please select the one below that is the best fit for your research. If you are not sure, read the appropriate sections before making your selection.

☒ Life sciences ☐ Behavioural & social sciences ☐ Ecological, evolutionary & environmental sciences

For a reference copy of the document with all sections, see nature.com/documents/nr-reporting-summary-flat.pdf

Life sciences study design

All studies must disclose on these points even when the disclosure is negative.

Sample size	All of 1,601 serum subjects and the associated clinical data were distributed from the NCGG Biobank, which collects human biomaterials and data for geriatrics research. Of them, 1,021 subjects were patients with AD: 91 patients with VaD, 169 patients with DLB, 32 patients with MCI and 288 subjects were normal controls with normal cognitive function (NC).
Data exclusions	The samples, no the status of the apolipoprotein E (APOE) genotype, no the Mini-Mental State Examination (MMSE) score, or <=60 years in age, were excluded from the analysis. All NC samples having a MMSE score <= 23 were also excluded from the analysis.
Replication	Data sets were divided into a discovery set and a validation (replication) set. All attempts at replication were successful.
Randomization	All participants were voluntary and would complete the informed consent in written before registering to our NCGG Biobank. Therefore, samples were randomly allocated into the experimental group.
Blinding	All samples were numbered and the investigators were blinded to the treatment group numbering system during data collection and analysis.

Reporting for specific materials, systems and methods

We require information from authors about some types of materials, experimental systems and methods used in many studies. Here, indicate whether each material, system or method listed is relevant to your study. If you are not sure if a list item applies to your research, read the appropriate section before selecting a response.

Materials & experimental systems

n/a	Involved in the study	
☒	☐	Antibodies
☒	☐	Eukaryotic cell lines
☒	☐	Palaeontology
☒	☐	Animals and other organisms
☐	☒	Human research participants
☒	☐	Clinical data

Methods

n/a	Involved in the study	
☒	☐	ChIP-seq
☒	☐	Flow cytometry
☒	☐	MRI-based neuroimaging

Human research participants

Policy information about studies involving human research participants

Population characteristics	Age, gender, the status of the apolipoprotein E (APOE) genotype.
Recruitment	All participants were voluntary and would complete the informed consent in written before registering to our NCGG Biobank.
Ethics oversight	This study was approved by the ethics committee of the National Center for Geriatrics and Gerontology (NCGG). The design and performance of current study involving human subjects were clearly described in a research protocol. All participants were voluntary and completed informed consent in writing before registering to NCGG Biobank.

Note that full information on the approval of the study protocol must also be provided in the manuscript.
