# Supplementary material for: Risk prediction models for dementia constructed by supervised principal component analysis using miRNA expression data
Source: Commun Biol. 2019 Feb 25;2:77. doi: 10.1038/s42003-019-0324-7 (PMC6389908; doi:10.1038/s42003-019-0324-7)
Supplement: Supplementary file 5 — Description of Additional Supplementary Files [file 42003_2019_324_MOESM5_ESM.docx]

**Description of Additional Supplementary Files**

**File Name**: Supplementary Data 1

**Description**: The list of AD-related miRNAs found in previous studies

**File Name**: Supplementary Data 2

**Description**: The list of miRNAs used for final risk prediction models
